# Improving children’s diets to address the double burden of malnutrition: a healthy diet is key for all

**DOI:** 10.1017/S1368980019002635

**Published:** 2019-09-20

**Authors:** Juana F Willumsen

**Affiliations:** Department for Prevention of Noncommunicable Diseases World Health Organization Avenue Appia 20, 1211 Geneva Switzerland Email: willumsenj@who.int

*‘Good nutrition allows children to survive, grow, develop, learn, play, participate and contribute.’*

*UNICEF, WHO and World Bank*^(1)^

However, with rapid urbanisation and changing patterns of food availability, food security and nutrition transition, overweight and obesity is rapidly becoming as pressing an issue as undernutrition and wasting. The double burden of malnutrition – whereby undernutrition, whether in the form of stunting, wasting or micronutrient deficiency, coexists with overweight and obesity or diet-related non-communicable diseases – can occur at the individual, household and population level and across the life course^(2)^. For example, a child who was born with low birth weight or was stunted during childhood is at greater risk of developing overweight or obesity in childhood or later life. In 2018, 40 million children under the age of 5 years were estimated to be overweight or obese and 49 million were wasted, the vast majority of them living in Asia and Africa^(1)^. Childhood obesity in older children affects 124 million^(3)^ and nearly one in five children aged 5–19 years is overweight or obese. Prevention for overweight and obesity needs to start even before conception^(4)^ and improving infant and young child feeding practices, from exclusive breast-feeding to complementary feeding and beyond, is key to protecting children from both undernutrition and overweight and obesity, making these critical double-duty actions. Double-duty actions are interventions or polices that can address both undernutrition (including wasting, stunting and micronutrient deficiency or insufficiency) and overweight, obesity or diet-related non-communicable diseases. Given the shared drivers of malnutrition in all its forms, such actions to tackle the double burden of malnutrition offer a unique opportunity to ensure nutrition interventions do no harm, are integrated and provide multiple benefits^(5)^. Addressing the double burden of malnutrition in young children will have multiple benefits, allowing children not only to survive but also to thrive and enabling them to make the most of the educational opportunities available to them.

The study by Williams *et al*. in this issue explores the potential of the Trials of Improved Practices methodology for improving the feeding and physical activity practices of caregivers of undernourished and overweight young children in Rwanda^(6)^. The use of this behaviour change technique to improve practices specifically for children with overweight is novel in this setting, where previously interventions have focused on undernutrition. In doing so, the authors identify not only many common young child feeding and physical activity practices that can be recommended for both underweight and overweight children, but also barriers to behaviour change that pose a particular issue for caregivers of overweight children. The belief that an overweight child is a healthy and happy child is prevalent and caregivers trying out different feeding practices received negative feedback from family and community members.

Helping caregivers identify when a child is under- or overweight and addressing beliefs about body size will be key to enabling improvements in infant diet. The WHO recently released guidance to assess children for overweight and obesity in primary health care in the context of the double burden of malnutrition^(7)^ and the Nurturing care framework^(8)^ highlights the importance of nutrition and caregiving that responds to children’s hunger and satiety. New guidelines on physical activity, sedentary behaviour and sleep for children under 5 years of age also provide recommendations on these behaviours in young children for their health and well-being^(9)^. However, all these initiatives and recommendations must be implemented in a comprehensive, integrated manner that supports caregivers and individual families, as well as the communities, cities and villages they live in, to promote not only optimal nutrition but also optimal care and development, so that no child is left behind and all children benefit from the best start in life. To do so, we will need to address not only the double burden of malnutrition, but also the wider questions of poverty, food insecurity and access to healthy diets, early childhood education and opportunities for physical activity, looking beyond the single issue through conversations for improved practices across sectors and societies.
